# Clinicopathological Prognostic Factors and Chemotherapeutic Outcome for Two Histopathological Types of Ampulla of Vater Adenocarcinoma

**DOI:** 10.3389/fonc.2021.616108

**Published:** 2021-02-18

**Authors:** Tao Xia, Xiaosan Wu, Yiping Mou, Yunyun Xu, Yucheng Zhou, Chao Lu, Qicong Zhu, Weiwei Jin, Yuan Chen

**Affiliations:** ^1^ Department of Gastrointestinal-Pancreatic Surgery, Zhejiang Provincial People’s Hospital, Hangzhou, China; ^2^ Department of Surgery, Bengbu Medical College, Bengbu, China

**Keywords:** adenocarcinoma of the ampulla of Vater, pancreatobiliary type, intestinal type, chemotherapy, CA199, carcinoembryonic antigen

## Abstract

**Background:**

Adenocarcinoma of the ampulla of Vater (AAV) is standardly treated using a complex operation, a pancreatoduodenectomy (PD), to remove the tumor. However, dicision-making in AAV clinical treatment remains difficult due to the broad range of AAV types, outcomes, and responses to special chemotherapeutics. Thus, this study aimed to explore clinicopathological prognostic factors associated with overall survival, as well as post-chemotherapeutic effects related to curative resection of AAV.

**Methods:**

We retrospectively reviewed data for clinicopathological outcome of 47 patients diagnosed with AAV that had underwent a PD. Overall survival probabilities were obtained using the Kaplan–Meier estimate method and a Cox proportional hazards model.

**Results:**

Forty-five patients underwent LPD (laparoscopic pancreatoduodenectomy) and two patients underwent PD. The patient group was composed of 31 males (66%) and 16 females (34%) with a mean age of 65(34–91)years. We selected 45 patients for long-term survival analysis. One- and three-year overall survival rates after resection were 97.6% and 58.9% respectively. The median survival was 37.7 months for the intestinal-type and 26.9 months in pancreatobiliary-type ampullary tumors. Serum carbohydrate antigen (CA) 19-9 greater than 37 U/ml (HR 0.140, P = 0.007), perineural invasion (HR 0.141, P = 0.003), and classification as pancreatobiliary-type (HR 6.633, P = 0.006) were independently associated with poor survival. Serum carcinoembryonic antigen (CEA) greater than 5 µg/ml (P = 0.031), serum CA 19-9 greater than 37 U/ml (P = 0.002), tumor sizes greater than 2.5cm (P=0.002), and positive perineural invasion (P=0.003) were all associated with a poor prognosis in the histopathological subgroup. Serum CA 19-9 greater than 37 U/ml (P=0.002) and positive perineural invasion (P=0.001) were significantly associated with poor survival in of patients with intestinal-type ampullary tumors. Serum CEA greater than 5 µg/ml (P=0.013) and tumor sizes greater than 2.5cm (P=0.002) were significantly associated with poor survival in patients with pancreatobiliary-type ampullary tumors.

**Conclusions:**

Pancreatobiliary-type ampullary tumors were associated with poor survival. Serum CA 19-9 in the intestinal-type and CEA in the pancreatobiliary-type were significantly associated with poor survival. Ajuvant chemotherapy could not predict the survival of AAV patients.

## Introduction

The ampulla of Vater comprises the major papilla, a common channel connecting the distal bile duct and main pancreatic duct to the duodenum. Adenocarcinoma of the ampulla of Vater (AAV) is the second most common malignancy of the periampullary region, which is treated using curative-intent pancreatoduodenectomy (PD) (1). Histopathologic studies have further characterized ampullary carcinoma as intestinal- or pancreaticobiliary-type based on the epithelium of origin. Intestinal-ampullary adenocarcinomas originate from the intestinal epithelium overlying the ampulla, whereas pancreaticobiliary-ampullary adenocarcinomas originate from the epithelium of the distal bile duct and distal pancreatic duct (2). Previous assessments have suggested that the prognosis of AAV is mainly correlated to the histopathologic differentiation of the two known types. However, decision-making remains hampered in clinical treatment due to the broad range of outcomes of different the AAV types and responses to chemotherapeutics post PD (3). To address the issue in AAV treatment, the present study aimed to explore better clinicopathological prognostic factors associated with overall survival, as well as post-chemotherapeutic effects related to curative resection of AAV.

## Patients and Methods

### Patients

We retrospectively reviewed data for the clinicopathological outcome of 47 patients diagnosed with AAV who underwent a PD between March 2015 and December 2019 from Zhejiang Provincial People’s Hospital. The hospital ethics committee gave approval for this data acquisition. All patients who underwent a curative-intent PD were enrolled in the study, however, we excluded patients with stage IV disease at diagnosis. Notably, 45 patients underwent laparoscopic pancreatoduodenectomy (LPD), whereas two patients had been subjected to PD. Genderwise, there were 31 males (66%) and 16 females (34%), with a mean age of 65 (34–91) years and mean BMI of 22.24 (16.53–29.41) kg/m^2^.

### Histopathological Evaluation and Immunohistochemistry

**Figure 1:**
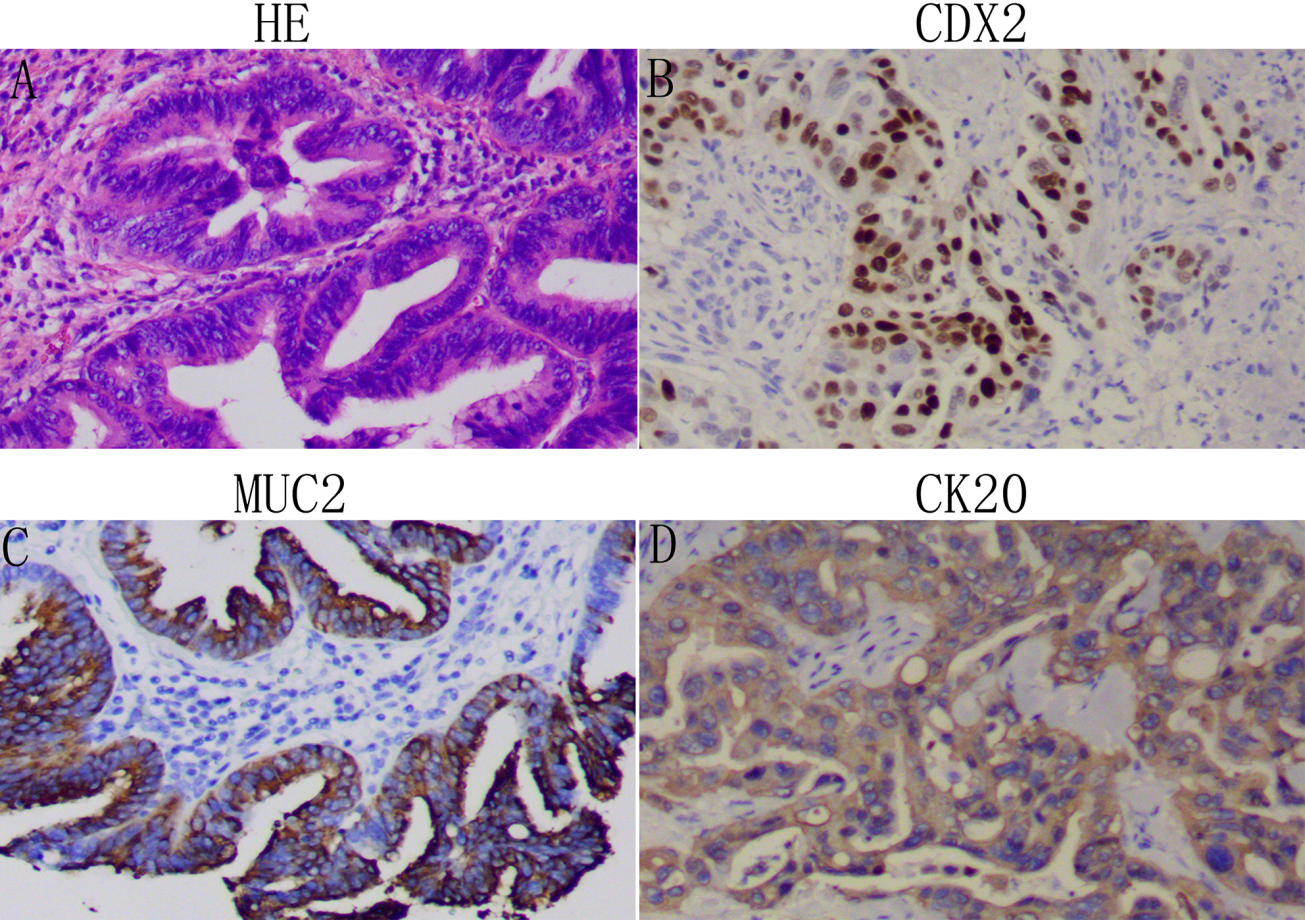
Identification of the intestinal-type adenocarcinoma of the ampulla of Vater (AAV): Representative images of patient tissue, HE **(A)**. Immunostaining for the intestinal-type markers CDX2 **(B)**, MUC2 **(C)**, and CK20 **(D)**. Magnification: 100x.

**Figure 2:**
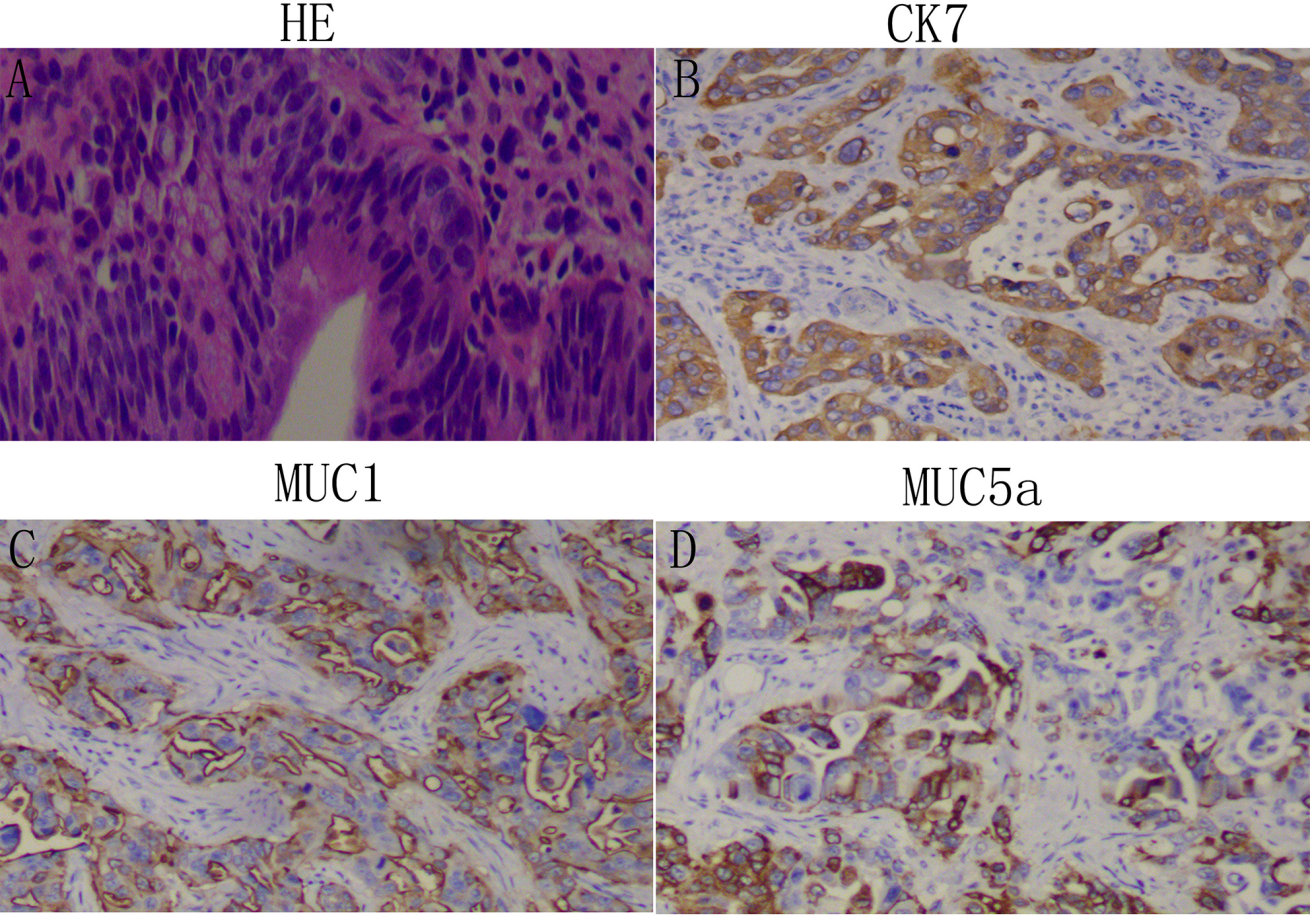
Identification of the pancreaticobiliary-type adenocarcinoma of the ampulla of Vater (AAV): Representative images of patient tissue, HE **(A)**. Immunostaining for the pancreaticobiliary-type markers Ck7 **(B)**, MUC1 **(C)**, and MUC5a **(D)**. Magnification: 100x.

Two histopathology specialists independently verified AAV diagnoses and their histopathological types. Classification criteria for intestinal- or pancreatobiliary-type AAV were conducted as described by Kimura et al. (2) and using molecular markers defined by Chang et al. (3). All immunohistochemistry analysis was performed on 4 µm thick serial sections of resected patient tissue. Expression of Caudal homeobox gene transcription factor 2 (CDX2), cytocheratin (CK)20, CK7, and apomucins (MUC2, MUC1, MUC5a) were verified in the validation cohorts. The intestinal-type AAV is, in most cases, known to express CDX2, CK20, MUC2 (**Figure 1**), whereas the pancreatobiliary-type AAV is determined by positive immunostaining for MUC1, MUC5a, and CK7 (**Figure 2**). Staging was performed according to the American Joint Committee on Cancer (AJCC, 8^th^ edition) (4).

### Statistical Analysis

SPSS v.22.0 and GraphPad Prism 8 software were used for statistical analyses. The Student’s t-test, Chi-square test, or Fisher’s exact test were used to analyze categorical variables, as appropriate. Overall survival probabilities were obtained using the Kaplan–Meier estimate method, and comparisons were conducted using the log-rank test. Risk factors with a p-value less than 0.05 in univariate models were tested in a Cox proportional hazards model. P-values less than 0.05 were considered statistically significant.

## Results

Seven patients had a postoperative hemorrhage, and four patients underwent reoperation. Two patients were characterized by delayed gastric emptying, and two patients died perioperatively. The median length of postoperative stay was 15 (8-66) days. A summary of other clinical characteristics and morbidity of patients are summarized in **Table 1**.

**Table 1:** Demographic and clinical data (N=47).

Variables	
Sex	
Male	31 (66%)
Female	16 (34%)
Age (years)	65.30 ± 10.60
BMI (kg/m^2^)	22.24 ± 2.71
Complications	
Yes	28 (59.6%)
No	19 (40.4%)
Jaundice	
Yes	20 (42.6%)
No	27 (57.4%)
CEA	
>5 ug/ml	11 (23.4%)
≤5 ug/ml	36 (76.6%)
CA199	
>37 U/ml	21 (44.7%)
≤37 U/ml	26 (55.3%)
Operation	
LPD	45 (95.7%)
OPD	2(4.3%)
Clavien-Dindo	
I	11 (23.4%)
II	3 (6.3%)
IV	7 (15%)
V	2 (4.3%)
LOS (days)	22.94 ± 15.84

The mean tumor size was 2.58 (1–8.5) cm. Patient disease was classified based on the degree of differentiation: 13 low, 27 moderate, and 7 high. The proportion of patients with tumor sizes T1, T2, T3, and T4 was 19.1%, 48.9%, 25.5%, and 6.4% respectively. We revealed 11 (23.4%) patients with regional lymph node metastases, 20 (42.6%) patients with perineural infiltration, and 13 (27.7%) patients with vascular involvement. We identified the histopathologic subtypes of the 47 cases of carcinoma as 53.2% intestinal and 46.8% pancreatobiliary.

**Figure 3:**
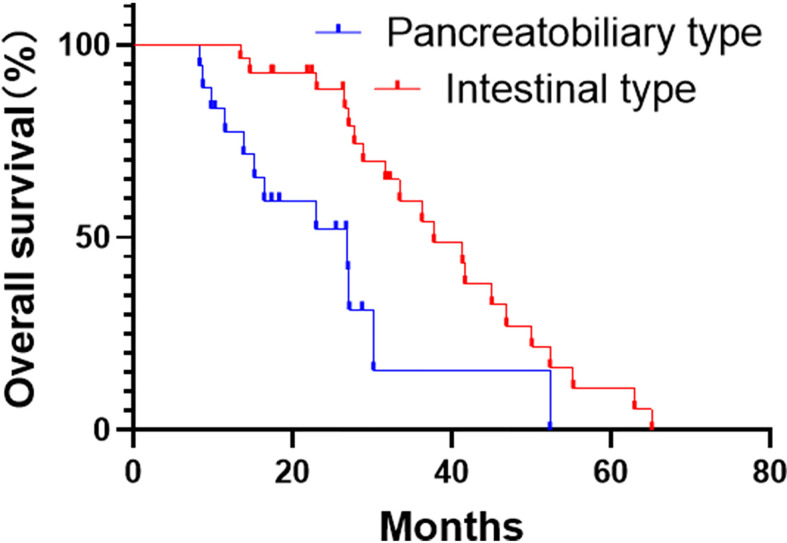
Kaplan-Meier curves for overall survival analysis of the pancreatobiliary- and intestinal-type adenocarcinoma of the ampulla of Vater (AAV).

Since two patients died perioperatively, only 45 patients were enrolled for long-term survival analysis. By the end of the study, the median follow-up period was 31.6 months. At that time, 13 patients died, and we lost contact with 1 patient. Of note, one and three-years overall survival rates after resection were 97.6% and 58.9% respectively. The median survival was 37.7 months in the intestinal-type and 26.9 months in the pancreatobiliary-type (**Figure 3**). Furthermore, 19 (42.2%) patients received postoperative, adjuvant chemotherapy. Oxaliplatin and capecitabine were administered as first-line chemotherapy for most of the patients; the remaining patients received a gemcitabine-based chemotherapy.

Through univariate analysis, we identified predictors of overall survival (**Table 2**). The cut-off value of serum CA 19-9 and CEA was determined according to the standard upper value. The cut-off value of tumor size was the median size of 2.5 cm. Notably, 5 prognostic factors were statistically significant, including jaundice (P=0.013), serum CA 19-9 > 37 U/ml (P=0.004), tumor sizes > 2.5 cm (P=0.017), positive perineural invasion (P=0.002), and classification as pancreatobiliary-type (P=0.046). Postoperative chemotherapy was not significantly associated with long-term survival (P=0.207). Using multivariable survival analysis, we revealed that serum CA 19-9 > 37 U/ml (HR 0.140, P=0.007), positive perineural invasion (HR 0.141, P=0.003), and the pancreatobiliary-type AAV (HR 6.633, P=0.006) were independently associated with poor survival.

**Table 2:** Univariate analysis and multivariable survival analysis (N = 45).

Variables		*P* value		HR	95% CI
		Univariate	Multivariate		
Sex		0.626			
Male	29				
Female	16				
Age (years)		0.628			
<60	15				
≥60	30				
BMI		0.228			
≤18.5	3				
18.5–24.99	35				
>25	7				
Jaundice		0.013	0.117		
Yes	19				
No	26				
CEA		0.073			
>5 ug/ml	11				
≤5 ug/ml	34				
CA199		0.004	0.007	0.140	0.034–0.578
>37 U/ml	21				
≤37 U/ml	24				
Size		0.017	0.025		
≤2.5 cm	29				
>2.5 cm	16				
Degree of differentiation		0.248			
Poor	12				
Moderate	26				
Well	7				
Perineural infiltration		0.002	0.003	0.141	0.039–0.508
Yes	20				
No	25				
Vascular involvement		0.789			
Yes	12				
No	33				
Stage		0.065			
I-II	34				
III	11				
Subtypes		0.046	0.006	6.633	1.735–25.362
Intestinal	27				
Pancreatobiliary	18				
Chemotherapy		0.207			
Yes	19				
No	26				

To validate the different prognoses of intestinal- and pancreatobiliary-type AAV, we independently evaluated prognostic factors in the subtypes of AAV. Interestingly, serum CEA > 5 µg/ml (P=0.031), serum CA 19-9 > 37 U/ml (P=0.002), tumor sizes > 2.5cm (P=0.002), and positive perineural invasion (P=0.003) were all associated with a poor prognosis in the histopathological subgroup of intestinal- and pancreatobiliary-type (**Table 3**). Furthermore, the outcomes for the intestinal- and pancreaticobiliary-type were similar with respect to gender, age, tumor size, differentiation, stage, vascular invasion, and perineural invasion.

**Table 3:** Prognostic factors in the two subtypes of AAV patients (N = 45).

Variables	Intestinal	Pancreatobiliary	P-value
Sex			0.401
Male	20 (74.1%)	9 (50%)	
Female	7 (25.9%)	9 (50%)	
Age (years)			0.724
<60	7 (25.9%)	8 (44.4%)	
≥60	20 (74.1%)	10 (55.6%)	
Jaundice			0.121
Yes	10 (37%)	9 (50%)	
No	17 (63%)	9 (50%)	
CEA			0.031
>5 ug/ml	7 (25.9%)	4 (22.2%)	
≤5 ug/ml	20 (74.1%)	14 (77.8%)	
CA199			0.002
>37 U/ml	11 (40.7%)	10 (55.6%)	
≤37 U/ml	16 (59.3%)	8 (44.4%)	
Size			0.002
≤2.5cm	17(63%)	12(66.7%)	
>2.5cm	10(37%)	6 (33.3%)	
Degree of differentiation			0.273
Poor	6 (22.2%)	6 (33.3%)	
Moderate	17 (63%)	9 (34.6%)	
Well	4 (14.8%)	3 (16.7%)	
Perineural infiltration			0.003
Yes	13 (48.1%)	7 (38.9%)	
No	14 (51.9%)	11 (61.1%)	
Vascular involvement			0.736
Yes	9 (33.3%)	3 (16.7%)	
No	18 (66.7%)	15 (83.3%)	
Stage 0			0.204
I-II	20 (74.1%)	14 (77.8%)	
III	7 (25.9%)	4 (22.2%)	

All the impact factors mentioned above had been introduced into each subgroup. Notably, CA 19-9 > 37 U/ml (P=0.002) and positive perineural invasion (P=0.001) were significantly associated with poor survival of patients with the intestinal-type ampullary tumors (**Table 4**). Additionally, serum CEA > 5 µg/ml (P=0.013) and tumor sizes > 2.5 cm (P=0.002) were significantly associated with poor survival in the pancreatobiliary-type ampullary tumors.

**Table 4:** Prognostic factors in the two AAV subtypes.

Variables	Intestinal	Pancreatobiliary
CEA		
>5 µg/ml	7 (25.9%)	4 (22.2%)
≤5 µg/ml	20 (74.1%)	14 (74.8%)
P-value	0.369	0.013
CA199		
>37 U/ml	11 (40.7%)	10 (55.6%)
≤37 U/ml	16 (59.3%)	8 (44.4%)
P-value	0.011	0.089
Size		
≤2.5 cm	17 (63%)	12 (66.7%)
>2.5 cm	10 (37%)	6 (33.3%)
P-value	0.125	0.000
Perineural infiltration		
Yes	13 (48.1%)	7 (38.9%)
No	14 (51.9%)	11 (61.1%)
P-value	0.001	0.318

## Discussion

Adenocarcinoma of the ampulla of Vater (AAV) is a rare cancer. It accounts for 6% and 0.2% of periampullary and gastrointestinal cancers respectively. A pancreatoduodenectomy (PD) is the standard curative treatment of ampulla adenocarcinoma, which has an intermediate overall survival between duodenal and pancreatic or distal bile duct cancer. Existing reports have revealed a 5-year survival rate ranging from 33% to 68%. The present study reviewed 45 patients that underwent a LPD and two patients that underwent a PD. Two patients died perioperatively, and one and three-years overall survival rates after resection were 97.6% and 58.9% respectively.

A wealth of studies (5, 6) have focused on the prognosis of ampullary adenocarcinoma. Different parameters, including depth of invasion (T stage), lymph node metastasis, poor differentiation, lymphovascular invasion, and perineural invasion have been revealed as prognostic factors for poor survival. Using univariate analysis, we found that total bilirubin, high serum CA 19-9, large tumor size, perineural invasion, and classification as the pancreatobiliary-type impacted the overall survival. In addition, the multivariate model demonstrated that high serum CA 19-9, perineural invasion, and the pancreatobiliary-types were independently associated with poor survival, a finding that concurs with previous reports (6–8). For instance, Vilhordo and colleagues (9) discovered the four prognostic factors of lymph node metastasis number, lymph node ratio (LNR), lymphovascularization, and differentiation grade of invasion. Numerous reports (10, 11) have also reported that the histological type is associated with overall survival, implicating poor prognosis for the pancreaticobiliary-type, as observed in our cohort.

Of note, AAV may originate from any of the three converge epithelia (duodenal, pancreatic, or biliary). Recent assessments have identified two distinct histological types of adenocarcinoma according to their original epithelium: intestinal and pancreatobiliary. Herein, the pathological subtypes of the 47 carcinomas were identified as the intestinal (53.2%) and pancreatobiliary types (46.8%). Elsewhere, Kim et al. found that pancreaticobiliary tumors were associated with a higher T stage, an advanced stage, and more significant risks of lymph node metastasis and perineural invasion. This could be a possible explanation as to why the prognosis for the pancreatobiliary-type is poorer (10). However, in the present study, the outcomes for the intestinal- and pancreaticobiliary-types were similar with respect to sex, age, jaundice, CEA, CA 19-9, tumor size, stage, differentiation, perineural invasion, vascular invasion, and stage, even with the poor prognosis of the pancreatobiliary-type. This finding is consistent with a few previously reported observations (7, 10, 12). Regarding survival analysis, serum CEA, serum CA 19-9, tumor sizes, and perineural invasion were all associated with a poor prognosis in the pathologic subgroup.

Furthermore, serum CA 19-9 and perineural invasion were significantly associated with poor survival in the intestinal-type ampullary tumors. Concurrently, serum CEA and tumor size were significantly associated with poor survival in the pancreatobiliary-type ampullary tumors. Interestingly, serum CA 19-9 in the intestinal-type and CEA in the pancreatobiliary-type were significantly associated with poor survival.

The role of adjuvant chemotherapy remains unknown for ampullary, distal bile duct and periampullary duodenal adenocarcinomas. In the ESPAC; study (13), 428 patients with ampullary adenocarcinomas were randomized to adjuvant chemotherapy (143 5-FU, 141 Gemcitabine) and 88 to observation. Notably, adjuvant chemotherapy was not associated with a significant survival benefit. However, multivariable analysis adjustment for prognostic variables demonstrated that adjuvant chemotherapy was significantly associated with a survival benefit. Although histological subtypes of ampullary adenocarcinoma have been identified, the interaction between the subtype and response to therapy is implicit. Interestingly, Ecker et al. (14) performed a multinational, retrospective cohort study in 12 institutions where patients with the pancreatobiliary subtype frequently received gemcitabine-based regimens, whereas patients with the intestinal subtype received more varied treatments (fluorouracil, 50.0%; gemcitabine, 44.1%). They reported no association between adjuvant therapy and overall survival, regardless of histological subtype. In this present study, 19 patients received adjuvant chemotherapy, but this did not predict the survival of patients with AAV.

There were some limitations to our present study. First, the study is in retrospective with a nonrandomized design; therefore, we could not interpret the associations as causative. Second, we could not explore 5-year survival rates because of short follow-up periods. Third, further analysis of the risk factors associated with chemotherapy in different types was impossible because of a small sample size.

In summary, this work found that serum CA 19-9 > 37 u/ml, positive perineural invasion, and classification as the pancreatobiliary type were prognostic factors independently associated with poor survival of AAV. Serum CA 19-9 > 37 U/ml and positive perineural invasion were significantly associated with poor survival in the intestinal-type ampullary tumors, whereas serum CEA > 5 µg/ml and tumor sizes > 2.5cm were significantly associated with poor survival in the pancreatobiliary-type ampullary tumors. Ajuvant chemotherapy, however, could not predict the survival of AAV patients. Future clinical studies aimed at evaluating adjuvant therapy for AAV should stratify the different subtypes to explore the potential benefit of treatment independently.

## Data Availability Statement

The original contributions presented in the study are included in the article/supplementary material. Further inquiries can be directed to the corresponding author.

## Ethics Statement

The studies involving human participants were reviewed and approved by the ethics committee of Zhejiang Provincial People’s Hospital. The patients/participants provided their written informed consent to participate in this study. Written informed consent was obtained from the individual(s) for the publication of any potentially identifiable images or data included in this article.

## Author Contributions

Conceptualization: YM and TX. Data curation: TX, XW, and YX. Formal analysis: TX. Investigation: XW, CL, and QZ. Methodology: TX, YC, and YZ. Resources: YM. Software: TX and WJ. Supervision: YM. Writing—original draft: TX, XW, and YM. Writing—review and editing: YM and, TX. All authors contributed to the article and approved the submitted version.

## Conflict of Interest

The authors declare that the research was conducted in the absence of any commercial or financial relationships that could be construed as a potential conflict of interest.

## References

[1] Kimura W Ohtsubo K. Incidence, sites of origin, and immunohistochemical and histochemical characteristics of atypical epithelium and minute carcinoma of the papilla of Vater. Cancer (1988) 61(7):1394–402. 10.1002/1097-0142(19880401)61:7<1394::AID-CNCR2820610720>3.0.CO;2-M 3422832

[2] Kimura W Futakawa N Yamagata S Wada Y Kuroda A Muto T. Different clinicopathologic findings in two histologic types of carcinoma of papilla of Vater. Jpn J Cancer Res Gann (1994) 85(2):161–6. 10.1111/j.1349-7006.1994.tb02077.x PMC5919425 7511574

[3] Chang DK Jamieson NB Johns AL Scarlett CJ Pajic M Chou A. Histomolecular phenotypes and outcome in adenocarcinoma of the ampulla of vater. J Clin Oncol Off J Am Soc Clin Oncol (2013) 31(10):1348–56. 10.1200/jco.2012.46.8868 23439753

[4] Amin MB Greene FL Edge SB Compton CC Gershenwald JE Brookland RK. The Eighth Edition AJCC Cancer Staging Manual: Continuing to build a bridge from a population-based to a more “personalized” approach to cancer staging. CA Cancer J Clin (2017) 67(2):93–9. 10.3322/caac.21388 28094848

[5] Winter JM Cameron JL Olino K Herman JM de Jong MC Hruban RH. Clinicopathologic analysis of ampullary neoplasms in 450 patients: implications for surgical strategy and long-term prognosis. J Gastrointest Surg Off J Soc Surg Alimentary Tract (2010) 14(2):379–87. 10.1007/s11605-009-1080-7 19911239

[6] Albores-Saavedra J Schwartz AM Batich K Henson DE. Cancers of the ampulla of vater: demographics, morphology, and survival based on 5,625 cases from the SEER program. J Surg Oncol (2009) 100(7):598–605. 10.1002/jso.21374 19697352

[7] Carter JT Grenert JP Rubenstein L Stewart L Way LW. Tumors of the ampulla of vater: histopathologic classification and predictors of survival. J Am Coll Surg (2008) 207(2):210–8. 10.1016/j.jamcollsurg.2008.01.028 18656049

[8] Qiao QL Zhao YG Ye ML Yang YM Zhao JX Huang YT. Carcinoma of the ampulla of Vater: factors influencing long-term survival of 127 patients with resection. World J Surg (2007) 31(1):137–43; discussion 44-6. 10.1007/s00268-006-0213-3 17171495

[9] Vilhordo DW Gregório C Valentini DF Jr. Edelweiss MIA Uchoa DM Osvaldt AB. Prognostic Factors of Long-term Survival Following Radical Resection for Ampullary Carcinoma. J Gastrointest Cancer (2020). 10.1007/s12029-020-00479-9 32808236

[10] Kim WS Choi DW Choi SH Heo JS You DD Lee HG. Clinical significance of pathologic subtype in curatively resected ampulla of vater cancer. J Surg Oncol (2012) 105(3):266–72. 10.1002/jso.22090 21882202

[11] Zimmermann C Wolk S Aust DE Meier F Saeger HD Ehehalt F. The pathohistological subtype strongly predicts survival in patients with ampullary carcinoma. Sci Rep (2019) 9(1):12676. 10.1038/s41598-019-49179-w 31481741 PMC6722235

[12] Kurihara C Yoshimi F Sasaki K Iijima T Kawasaki H Nagai H. Clinical value of serum CA19-9 as a prognostic factor for the ampulla of Vater carcinoma. Hepato-Gastroenterology (2013) 60(127):1588–91. 10.5754/hge13150 23933785

[13] Neoptolemos JP Moore MJ Cox TF Valle JW Palmer DH McDonald AC. Effect of adjuvant chemotherapy with fluorouracil plus folinic acid or gemcitabine vs observation on survival in patients with resected periampullary adenocarcinoma: the ESPAC-3 periampullary cancer randomized trial. Jama (2012) 308(2):147–56. 10.1001/jama.2012.14674 22782416

[14] Ecker BL Vollmer CM Jr. Behrman SW Allegrini V Aversa J Ball CG. Role of Adjuvant Multimodality Therapy After Curative-Intent Resection of Ampullary Carcinoma. JAMA Surg (2019) 154(8):706–14. 10.1001/jamasurg.2019.1170 PMC6547142 31141112

